# Effect of Postharvest Storage Temperature and Duration on Tomato Fruit Quality

**DOI:** 10.3390/foods14061002

**Published:** 2025-03-15

**Authors:** Xueou Li, Huofeng Huang, Lida Zhang, Lingxia Zhao

**Affiliations:** 1 Department of Plant Science, School of Agriculture and Biology, Shanghai Jiao Tong University, 800 Dongchuan Road, Shanghai 200240, China; xueouli@sjtu.edu.cn (X.L.); 13804807105@163.com (H.H.); zhangld@sjtu.edu.cn (L.Z.); 2 Joint Tomato Research Institute, School of Agriculture and Biology, Shanghai Jiao Tong University, 800 Dongchuan Road, Shanghai 200240, China

**Keywords:** tomato fruit, low temperature, storage period, quality attributes

## Abstract

Tomato (*Solanum lycopersicum*), a leading vegetable crop of significant economic importance, is a valuable source of nutrients and minerals in the human diet. Consumer and breeder interest focuses extensively on tomato quality attributes, including appearance, texture, flavor, and nutritional value. While moderate low temperatures are generally beneficial for preserving tomato quality during transportation and storage, the precise effects of storage temperature on these qualities remain to be fully elucidated. This study investigated the changes in quality attributes of tomato (cv. Shangjiao No.2) fruit stored at different temperatures (4 °C, 14 °C, and 24 °C) for varying durations (0, 1, 5, 9, and 15 days postharvest, dph). Results showed that low temperatures (4 °C and 14 °C) were beneficial for maintaining fruit appearance and total soluble solids (TSS) content. Furthermore, 4 °C storage effectively delayed ascorbic acid (Vitamin C) loss. Storage at both 4 °C and 14 °C similarly and significantly reduced fruit softening and water loss rate (WLR). This reduction was associated with the temperature-regulated expression of cell wall-related genes, including *SlCESA6*, *SlCEL2*, *SlEXP1*, and *SlPL*. The activities of cell wall-degrading enzymes, such as polygalacturonase (PG), β-galactosidase (β-Gal), and cellulase, were also significantly inhibited at lower storage temperatures. Additionally, storage at 24 °C caused considerable damage to plastid ultrastructure. Although temperature had a minor effect on carotenoid, the reduction in carotenoid levels was less pronounced at 4 °C. While low-temperature storage suppressed the release of some aroma compounds, it also reduced the levels of undesirable volatiles. This study provides insights for optimizing storage temperature and duration to maintain tomato fruit quality.

## 1. Introduction

Tomato (*Solanum lycopersicum*) is the second most important vegetable crop globally [1]. Tomato fruits are prized for their vibrant color and rich content of bioactive compounds, including amino acids, minerals, and vitamins, which contribute to human health and offer protection against major diseases such as cardiovascular disorders [2]. Representing a major component of the human diet, tomatoes are broadly categorized into processing and fresh-market types [3,4]. Consequently, fruit quality is a central focus of tomato research and breeding efforts. Due to the spatial separation between production sites and consumer markets, maintaining the excellent quality of fruits during transportation and storage has become a critical aspect of tomato quality research.

Tomato quality is determined by a combination of factors, including appearance, texture, flavor, aroma, and nutritional value [5,6]. Fresh fruits and vegetables are primarily graded based on appearance [7], which encompasses fruit shape, color, and the degree of physical damage or pathogen infection. The defects compromise fruit integrity, reduce edibility, and ultimately severely impact nutritional value [8]. Ascorbic acid (AsA) is an important vitamin in tomatoes and a key indicator of their nutritional quality. However, AsA is prone to oxidation into dehydroascorbic acid during storage, leading to a loss of its biological activity [9]. The rate of AsA decline is influenced by storage temperature, harvest time, and is generally positively correlated with the maturity of the tomato [10].

Firmer tomatoes are better able to maintain their shape and have an extended shelf life. Fruit ripening in tomatoes is accompanied by softening, a process regulated by cell wall-degrading or -modifying enzymes. During postharvest storage of apricot (*Prunus armeniaca*) fruit, increased polygalacturonase (PG) activity and elevated expression of the *PaPG1* gene are associated with fruit softening and ethylene production [11,12]. Furthermore, transgenic strawberry plants with silenced *FaPG1* exhibit delayed fruit softening [13]. These findings demonstrate that PG plays a crucial role in fruit softening. β-Galactosidase (β-Gal) is capable of degrading pectin and cell wall structure, and its enzymatic activity shows a negative correlation with fruit firmness [14]. Cellulase, which specifically cleaves glucan chains, is a major enzyme contributing to the softening of fruits such as mango (*Mangifera indica* L.) [15] and guava (*Psidium guajava*) [16]. Temperature significantly influences the activity of these enzymes [17], making appropriate storage temperature a crucial factor in maintaining fruit firmness.

Carotenoids, a vital class of terpenoid compounds, determine the color of tomato fruit and are major nutritional components of ripe fruit [18]. Studies have shown that postharvest storage can promote carotenoid accumulation, potentially due to continued biosynthesis with reduced turnover and/or enhanced sequestration [19]. The accumulation of carotenoids in postharvest fruit is primarily related to temperature [20]. At a storage temperature of 12 °C, changes in pigment accumulation occur in grapefruit, characterized by chlorophyll degradation and linear carotenoid accumulation. The cessation of chilling injury symptom development after three weeks of storage is correlated with an increase in linear carotenoids [21,22]. Research indicates that lycopene, particularly phytoene and phytofluene, exhibits significant free radical scavenging properties, protecting plastid structures from reactive oxygen species [23]. Three key genes, *SlPSY1* (*PHYTOENE SYNTHASE 1*), *SlCRTISO* (*CAROTENOID ISOMERASE*), and *SlCYCB* (*CHROMOPLAST SPECIFIC LYCOPENE Β-CYCLASE*), play crucial roles in the carotenoid biosynthesis pathway [24]. SlPSY1 catalyzes the condensation of two molecules of GGPP (Geranylgeranyl Diphosphate) derived from the MEP (Methylerythritol Phosphate) pathway into phytoene, representing the rate-limiting step in the initial phase of the pathway. *SlCRTISO* encodes an isomerase that converts cis-(prelycopene)/trans-carotenoids (all-trans-lycopene) in the linear carotenoid pathway, determining the synthesis of lycopene and β-carotene branch products [25]. It also influences the biosynthesis of abscisic acid (ABA) and strigolactones, serving as a key enzyme in the synthesis of functional products in the carotenoid pathway [26]. *SlCYCB* encodes a chromoplast-specific cyclase that cyclizes lycopene, a crucial enzyme determining the synthesis of cyclic carotenoids [27].

The composition and content of volatile compounds influence the aroma and flavor of tomato fruit [28]. Storage temperature and fruit pre-treatments significantly affect the levels of volatile compounds [29]. Studies have shown that low-temperature storage reduces the levels of major volatile compounds in tomatoes, and the transcript levels of several genes encoding key volatile synthesis enzymes, whereas, an effect was not observed during storage at higher temperatures [30]. Similarly, postharvest heat treatments can also influence the release of volatile compounds. For instance, the release of ester compounds, which are crucial for apple aroma, is inhibited by heat treatment [31]. These observations suggest that the biosynthesis pathways of volatile compounds during fruit storage are primarily dependent on enzymatic catalysis. Therefore, factors such as temperature and pre-treatments indirectly influence the production and release of volatile compounds by affecting enzyme activity, ultimately impacting the overall flavor of tomatoes.

Therefore, this study used Shangjiao No.2 (purple fruit) tomatoes to investigate the effects of temperature (4 °C, 14 °C, and 24 °C) and storage duration (0, 1, 5, 9, and 15 dph) on postharvest physiology and quality. We analyzed the appearance, firmness, water loss, flavor components (total soluble solids, volatile compounds), ascorbic acid content, and carotenoid content of tomato fruits stored at different temperatures. Furthermore, this study explored the effects of temperature on tomato fruit quality at different storage periods from anatomical (plastid development and exocarp microstructure), gene transcriptional (carotenoid synthesis pathway and firmness-related gene expression), and cell wall-related enzyme activity levels. The aim was to determine the optimal storage temperature and duration for Shangjiao No.2 tomatoes, providing a theoretical basis for guiding tomato fruit storage and preservation, quality regulation, and consumer purchasing decisions.

## 2. Materials and Methods

### 2.1. Plant Material and Growth Conditions

The cultivated tomato (Shangjiao No.2, purple fruit) used in this experiment is a new variety through distant hybridization using “heirloom” resources introduced from North America. The plants were grown in a standard greenhouse at the Shanghai Jiao Tong University Pujiang Green Valley (121°30′9″ E; 31°3′5″ N). The naturally ripening fruits were harvested in the morning and placed at room temperature for 2 h. Tomato fruits with no pests, diseases, or mechanical damage, and with uniform size, weight, and maturity, were selected and stored at 4 °C, 14 °C, and 24 °C. Samples were taken for analysis at 0, 1, 5, 9, and 15 dph.

### 2.2. Fruit Firmness and Water Loss Rate

Fruit firmness was measured using a TA-XTplus texture analyzer (Stable Micro System, Surrey, UK) equipped with a 5 cm cylindrical probe. Firmness was measured at the top and equator of the tomato fruit (unit: N) with five biological replicates. The water loss rate (WLR, three biological replicates) was calculated by the formula: WLR (%) = (W_0_ − W_n_)/W_0_ × 100%; W_0_ indicates fruit weight on the harvest day; Wn indicates the fruit weight after storage; and *n* = 1, 5, 9, 15.

### 2.3. Total Soluble Solids Content

The total soluble solids (TSS) content of tomatoes was measured using a handheld refractometer (ATAGO, Tokyo, Japan) with three biological replicates. Tomato fruits were homogenized using a blender (Philips, Amsterdam, The Netherlands), and the homogenate was squeezed through cheesecloth. The homogenate was then dripped onto the glass surface of the refractometer, and the data were measured and recorded. The refractometer was cleaned, calibrated, and zeroed with ddH_2_O before each measurement.

### 2.4. Ascorbic Acid Content

The ascorbic acid content was measured according to previous research [32]. For calibration, 1 mL of AsA (Sangon Biotech, Shanghai, China) standard solution (1 g/mL) was added to 10 mL of oxalic acid (Sangon Biotech, Shanghai, China) solution (2%) and mixed thoroughly (the control was performed using 2% oxalic acid solution). The mixture was titrated with 2,6-dichloroindophenol (Sinopharm Chemical Reagent Co., Ltd., Shanghai, China) solution (0.2 g/L) until a stable pink color (lasting for at least 15 s) was achieved. The volume of titrant used was then recorded and three replicates were performed. The titer was calculated according to the formula T = C × V/(V_1_ − V_0_), where T represents ascorbic acid content (mg)/2,6-dichloroindophenol solution (mL); C represents the concentration of ascorbic acid standard solution (mg/mL); V represents AsA standard solution volume; and V_1_ and V_0_ represent 2,6-dichloroindophenol solution volumes consumed for titrating the AsA standard solution and the control (mL), respectively.

A total of 20 g of tomato fruit samples was homogenized with an equal weight of 2% (*w*/*v*) oxalic acid solution. Subsequently, 20 g of the homogenate was transferred to a 100 mL volumetric flask and brought to volume with the oxalic acid solution. After thorough mixing, 40 g of this solution was placed in a centrifuge tube, and 3 g of kaolin was added. The mixture was then vortexed and centrifuged at 4 °C at 4000× *g* for 5 min. A 10 mL volume of the supernatant was titrated with 2,6-dichloroindophenol solution until a persistent pink was reached and maintained for at least 15 s. Following three biological replicates, the ascorbic acid content was calculated using the formula X = (V − V_0_) × T × A/m, where X represents ascorbic acid content (mg/100 g); V and V_0_ represent volumes of 2,6-dichloroindophenol solution used for sample and control titration, respectively (mL); T indicates ascorbic acid content (mg)/2,6-dichloroindophenol solution (mL); A is dilution ratio; and m is sample weight (g).

### 2.5. Anatomical Analysis

The pericarp tissue of about 1 mm^3^ in size was cut on the equatorial plane of the fruit, and quickly put into 2.5% glutaraldehyde (Zhongjingkeyi Technology Co., Ltd, Beijing, China) fixative. Then, the sample was placed in a vacuum drying oven for 30 min until the tissue was completely immersed, and then placed in a 4 °C refrigerator to fix for 24 h. After discarding the fixative, 0.1 M PB solution (Sangon Biotech, Shanghai, China) was added to wash the sample, and then placed in a 4 °C refrigerator for 15 min, repeated 4 times. After discarding the washing solution, 0.2 mL of fixative was added and placed in a 4 °C refrigerator for 1.5 h, the fixative replaced with ultrapure water, and placed in a 4 °C refrigerator for 10 min, repeated 2 times. Subsequently, the samples were dehydrated by gradient ethanol (50%, 70%, 90%) for 15 min, respectively, and the samples were replaced with 90% ethanol:90% acetone (1:1), 90% acetone and 90% acetone for 20 min, and 100% acetone for 3 times, each for 10 min. Next, acetone:epoxy resin with a volume ratio of 1:1, 1:2, 1:3 and pure resin were used to replace the acetone in the samples, for 1 h, 1 h, 12 h, and 3 h, respectively. The samples were carefully placed in an embedding plate and placed at 60 °C for 48 h. The sections were sliced from the resin block and stained with osmic acid. The ethanol, acetone, and osmic acid were purchased from Sinopharm Chemical Reagent Co., Ltd. (Shanghai, China), and resin was sourced from Ted Pella Inc. (Redding, CA, USA). The ultrastructure of chromoplasts was observed using a 120 kV biological transmission electron microscope (Thermofisher, Waltham, MA, USA), and the microstructure of fruit pericarp cells was observed using a Leica DM6B microscope (Leica, Nussloch, Germany).

### 2.6. Carotenoid Content

Tomato carotenoid extraction was performed following a previously described method [33]. Fruit samples were frozen in liquid nitrogen and ground to a homogeneous powder. All subsequent extraction steps were performed under light-protected conditions, with three biological replicates. The reagents utilized in this experiment were sourced from Sinopharm Chemical Reagent Co., Ltd. (Shanghai, China). A mass of 0.5 g of fruit powder was weighed into a centrifuge tube, 1.5 mL of methanol and 200 μL of potassium hydroxide (60%, *w*/*v*) were added, and the mixture was vortexed for 3 min. The centrifuge tube was then incubated in a 60 °C water bath for 30 min, with mixing 2–3 times during incubation, followed by cooling to room temperature. A volume of 1.5 mL of Tris-NaCl buffer (50 mM Tris-HCl, 1 M NaCl, pH 7.5) was added, and the mixture was inverted to mix, then incubated at 4 °C for 10 min, with mixing 1–2 times. Then, 4 mL of chloroform (4 °C) was added, and the sample was gently mixed on ice for 10 min, followed by centrifugation at 4000 rpm for 10 min at 4 °C. The lower chloroform phase was carefully collected using a 5 mL syringe and transferred to a new centrifuge tube, avoiding any contamination from the interface or upper phase. The aqueous phase was then re-extracted with 4 mL of chloroform. The chloroform extracts were combined and brought to a final volume of 10 mL with chloroform.

A total of 1 mL of the combined extract was transferred to a centrifuge tube and dried in a vacuum concentrator for 6 h with the lid open. The residue was reconstituted in 50 μL of methyl tert-butyl ether (MTBE), vortexed thoroughly, and centrifuged at 12,000 rpm for 10 min at 4 °C. Then, 20 μL of the supernatant was transferred to a vial for analysis. Ultra-performance convergence chromatography (UPC2) parameters were set according to a previous study [34]. Standard curves were generated using lycopene, α-carotene, β-carotene, and lutein solutions at concentrations of 5 μg/mL, 10 μg/mL, 50 μg/mL, 250 μg/mL, and 500 μg/mL.

### 2.7. Volatile Compounds

The volatile compounds of tomato fruit were determined using a solid-phase microextraction-gas chromatograph-mass spectrometer (SMPE-GC-MS) method with reference to previous studies and with slight modifications [35]. A total of 1 g of tomato sample was taken into a 20 mL headspace vial, crushed into a homogenate with a glass rod, 10 μL of 2-octanol (Sinopharm Chemical Reagent Co., Ltd., Shanghai, China) standard solution (10 μg/mL) was added, the lid was tightened, and the machine was tested.

The aroma characteristic compounds and internal standard substances of tomato were qualitatively analyzed by the NIST library. The content of the target substance was calculated by peak area of target substance × internal standard concentration/peak area of internal standard.

### 2.8. Reverse Transcription-Quantitative PCR (RT-qPCR)

Total RNA was extracted from tissues ground into fine powder in liquid nitrogen using the RNAprep Pure Plant Kit (Tiangen, Beijing, China). First-strand cDNA was synthesized from 1.0 μg of total RNA using the PrimeScript™ RT Master Mix kit (TAKARA, Dalian, China). The resulting cDNA library was diluted 50-fold and used as a template for qPCR analysis. qPCR was performed on a CFX96™ Real-Time System (Bio-Rad, Hercules, CA, USA) using Hieff qPCR SYBR Green Master Mix (Yeasen, Shanghai, China). The reaction (20 μL) contained 2 μL of diluted cDNA, 10 μL of SYBR Green Master Mix, 0.6 μL each of forward and reverse primers (10 μM) (Table S4), and 6.8 μL of RNase-free water. The qPCR cycling conditions were 94 °C for 3 min, followed by 40 cycles of 94 °C for 20 s, 55 °C for 20 s, and 72 °C for 20 s. Relative transcription levels were calculated using the 2^−ΔΔCT^ method, with *SlACTIN2* (Solyc11g005330) as the normalized internal reference gene [36].

### 2.9. Cell Wall-Related Enzyme Activity Measurement

A total of 2 g of tomato pericarp powder was mixed with 1 mL of pre-cooled 80% ethanol, and centrifuged at 12,000× *g* for 10 min, and the supernatant discarded. A volume of 1 mL of extraction solution (50 mmol/L, pH 5.5 sodium acetate buffer and 1.8 mol/L NaCl) was added to the precipitate, stirred for 20 min and centrifuged again, and the supernatant was the enzyme extraction solution. The above process was carried out at 4 °C. Additionally, 0, 0.2, 0.4, 0.6, 0.8, 1.0, and 1.2 mg/mL glucose solutions were prepared to make a standard curve for cellulase and PG activity calculation, with 0, 0.2, 0.4, 0.8, 1.2, 1.6, 2, 2.4, 2.8, and 3 mmol/L p-nitrophenol solution for β-Gal activity calculation. Analytical standards were purchased from Aladdin Biochemical Technology Co., Ltd. (Shanghai, China), and the other reagents were obtained from Sinopharm Chemical Reagent Co., Ltd. (Shanghai, China).

A volume of 5 mL of enzyme extract (equal amount of enzyme solution was boiled for 5 min and added to the control) was added to 1.5 mL 10 g/L carboxymethyl cellulose sodium solution, and incubated in a 37 °C water bath for 1 h. Then, 1.5 mL of 3,5-dinitrosalicylic acid reagent was quickly added and heated in a boiling water bath for 5 min, quickly cooled to room temperature, and mixed well. We measured the absorbance value of the solution at a wavelength of 540 nm to calculate cellulase activity.

Volumes of 1 mL of 50 mmol/L sodium acetate buffer (pH 5.5) and 1 mL of 1% polygalacturonic acid solution were added to 0.5 mL of enzyme extract (equal amount of enzyme solution was boiled for 5 min and added to the control), respectively. After mixing, the mixture was placed in a 37 °C water bath for 1 h, then 1.5 mL of 3,5-dinitrosalicylic acid reagent was quickly added and heated in a boiling water bath for 5 min, quickly cooled to room temperature, and mixed well. The absorbance value of the solution was measured at a wavelength of 540 nm to calculate PG activity.

A volume of 0.9 mL of 5 mmol/L p-nitrophenyl-β-D-galactopyranoside solution was added to 0.1 mL of enzyme extract (equal amount of enzyme solution was boiled for 5 min and added equal amount of Na_2_CO_3_ solution to the control), incubated in a 37 °C water bath for 30 min, removed, and 0.4 mL of 1 mol/L Na_2_CO_3_ solution was immediately added, mixed well, incubated in a 37 °C water bath for 30 min, and cooled to room temperature. The absorbance value of the mixture was measured at a wavelength of 400 nm to calculate β-Gal activity.

### 2.10. Data Statistics

Statistical analyses were performed using IBM SPSS version 25 (Statistical Product and Service Solutions, https://spss.en.softonic.com (accessed on 4 March 2020)), and a one-way ANOVA test was conducted to determine statistical significance at the *p* < 0.05 level.

## 3. Results

### 3.1. The Appearance, Soluble Solids Content, and Ascorbic Acid Content of Stored Tomato Fruit

**Figure 1:**
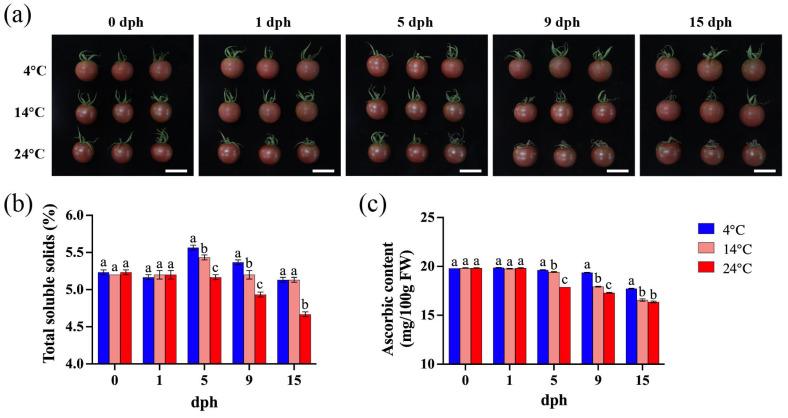
Tomato fruit appearance, soluble solids, and ascorbic acid content during storage (0, 1, 5, 9, 15 dph) at different temperatures (4, 14, 24 °C). (**a**) Fruit appearance, scale bar, 4 cm; (**b**) total soluble solids (%); (**c**) ascorbic content (mg/100 g FW). dph, days postharvest. FW, fresh weight. The data indicate averages ± standard deviations (SDs). Different lowercase letters indicate statistically significant differences (*p* < 0.05) as determined by a one-way ANOVA test.

The primary appearance difference in tomato stored at varying temperatures lies in the extent of sepal senescence. Storage at 4 °C preserves the sepal integrity with minimal change, even at 15 dph. In contrast, sepal wilting becomes apparent by 9 dph when stored at 14 °C. Furthermore, as storage temperature and duration increase, the sepal undergoes progressive darkening and increased wilting (Figure 1a).

Under 4 °C storage, the total soluble solids (TSS) content initially increased, peaked at 5 dph, and then decreased, reaching a level similar to the harvest day (0 dph) at 15 dph. The TSS content at 5 dph and 9 dph was significantly higher than that of tomato stored at 14 °C and 24 °C (Figure 1b). At 14 °C, the TSS content showed a slight increase at 5 dph, with minimal changes during other periods. Under 24 °C storage, the TSS content began to decline from 5 dph to 15 dph, and remained consistently lower than that observed at 4 °C and 14 °C (Figure 1b). These results indicate that temperature significantly influences TSS accumulation in tomato fruit.

The ascorbic acid (AsA) content consistently decreased from 5 to 15 dph, with a faster rate of decline observed at higher temperatures (Figure 1c). Between 5 and 15 dph, tomatoes stored at 4 °C exhibited significantly higher AsA levels compared to those stored at higher temperatures (Figure 1c). In contrast, during the initial period (0–5 dph), ascorbic acid levels remained relatively high and stable across all storage temperatures, showing no significant differences (Figure 1c). Consequently, low-temperature storage is beneficial for maintaining AsA content in tomato fruit.

### 3.2. Tomato Fruit Firmness and Cell Wall Metabolism During Storage

**Figure 2:**
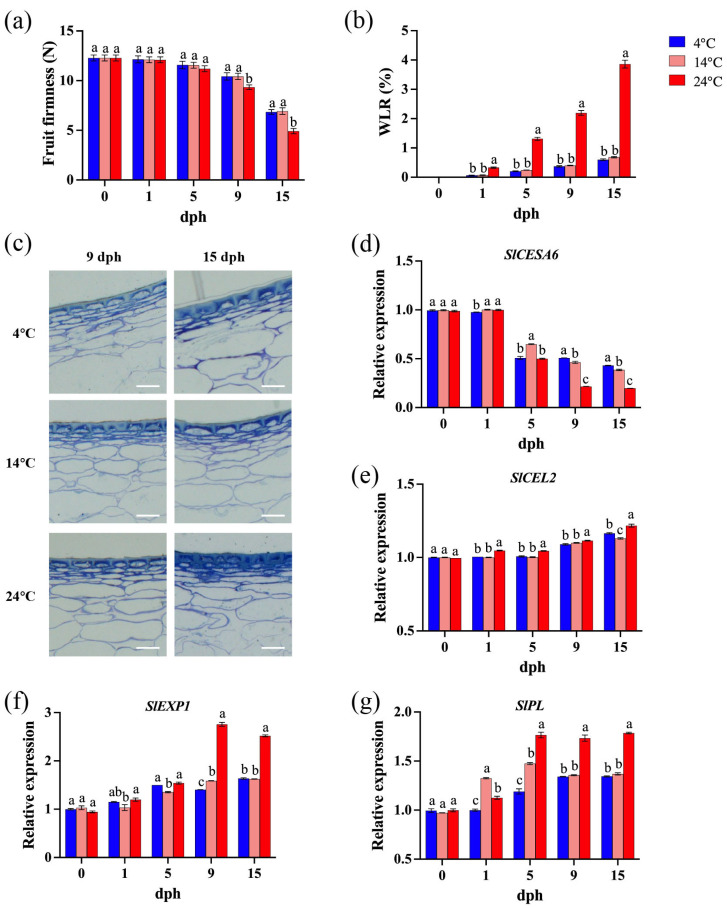
Tomato pericarp cell wall metabolism during storage (0, 1, 5, 9, 15 dph) at different temperatures (4, 14, 24 °C). (**a**) Fruit firmness (N); (**b**) water loss rate (WLR, %); (**c**) pericarp cell wall structure of 9 dph and 15 dph. Scale bar, 100 μm. (**d**) Relative expression of *SlCESA6* gene; (**e**) relative expression of *SlCEL2* gene; (**f**) relative expression of *SlEXP1* gene; (**g**) relative expression of *SlPL* gene. dph, days postharvest. The data indicate averages ± standard deviations (SDs). Different lowercase letters indicate statistically significant differences (*p* < 0.05) as determined by a one-way ANOVA test.

Fruit firmness is a key determinant of postharvest shelf life and commercial value in tomato [37]. Transpiration in fruits and vegetables leads to tissue dehydration, shriveling, and softening, resulting in a loss of freshness [38]. During the initial 0–5 dph, storage temperature and time had minimal impact on tomato fruit firmness. However, as time progressed (9–15 dph), elevated storage temperatures accelerated fruit softening (Figure 2a). Specifically, tomatoes stored at 24 °C exhibited significantly lower firmness compared to those stored at lower temperatures. While fruit softening continued at both 4 °C and 14 °C, no significant difference in firmness was observed between these two lower temperatures (Figure 2a). Fruit water loss increased progressively, and storage temperature significantly impacted the postharvest water loss rate (WLR) of tomato; specifically, at 24 °C, the WLR was consistently and significantly higher than at lower temperatures (Figure 2b). Extended storage duration and elevated temperatures lead to progressive wrinkling and the degradation of integral cellular morphology in tomato pericarp (Figure 2c). These findings indicate that storage at 24 °C is detrimental to delaying the softening and prolonging shelf life of tomato fruit.

Tomato fruit softening is closely associated with the depolymerization of cell wall polysaccharides and a reduction in intercellular adhesion [39]. *SlCESA6* (*CELLULOSE SYNTHASE 6*), involved in primary cell wall biosynthesis and cellulose synthesis [40], is hypothesized to contribute to increased fruit firmness. Transcript levels of *SlCESA6* decreased during postharvest storage, with a more pronounced decline observed in fruit stored at 24 °C, especially at later stages (9–15 dph), compared to those stored at 4 °C and 14 °C (Figure 2d). The expression levels of *SlCEL2* (*CELLULASE 2*), *SlEXP1* (*EXPANSIN 1*), and *SlPL* (*PECTATE LYASE*) are correlated with fruit softening [39,40,41]. Generally, their transcript abundance increased with storage duration (Figure 2e–g). At 9 and 15 dph, *SlCEL2* and *SlEXP1* expression was also significantly higher at 24 °C compared to 4 °C and 14 °C (Figure 2e). Storage at 24 °C led to apparently and significantly higher transcript levels of *SlPL* compared to storage at 4 °C and 14 °C, particularly from 5 to 15 dph (Figure 2f,g).

**Figure 3:**
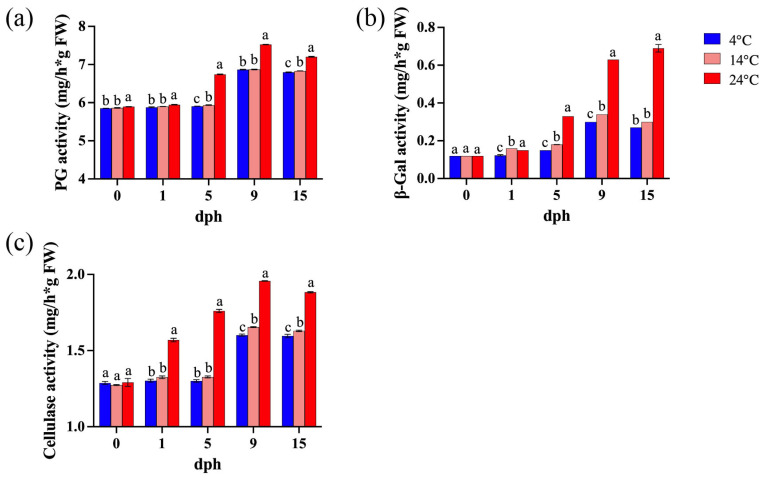
Enzyme activity in tomato fruit during storage (0, 1, 5, 9, 15 dph) at different temperatures (4, 14, 24 °C). (**a**) Polygalacturonase (PG) activity. (**b**) β-galactosidase (β-Gal) activity; (**c**) cellulase activity. dph, days postharvest. The data indicate averages ± standard deviations (SDs). Different lowercase letters indicate statistically significant differences (*p* < 0.05) as determined by a one-way ANOVA test.

Polygalacturonase (PG) degrades pectin polymers, leading to a loss of pectin viscosity and accelerated fruit softening [42]. β-galactosidase (β-Gal) degrades β-galactan, disrupting pectin structure and cell wall integrity by removing galactan side chains from pectin [43], and cellulase (CEL) weakens and loosens cell walls by cleaving β-1,4-glycosidic bonds [44]. The activity of cell wall-associated enzymes exhibited an increasing trend with prolonged storage time and elevated temperatures (Figure 3). During the 5–15 dph period, polygalacturonase (PG) activity showed an increasing trend, with significantly higher activity observed at 24 °C compared to lower storage temperatures. Although PG activity at 14 °C was only slightly higher than at 4 °C, the difference between them was statistically significant (Figure 3a). Similarly, storage at 24 °C resulted in a significant increase in β-galactosidase (β-Gal) activity, primarily during the 5–15 dph period, whereas the differences in β-Gal activity between 4 °C and 14 °C were relatively inconspicuous (Figure 3b). Elevated storage temperature (24 °C) significantly enhanced cellulase activity during the 1–15 dph period, while the differences between 4 °C and 14 °C were minor (Figure 3c).

### 3.3. Carotenoid Accumulation in Tomato Fruit

**Figure 4:**
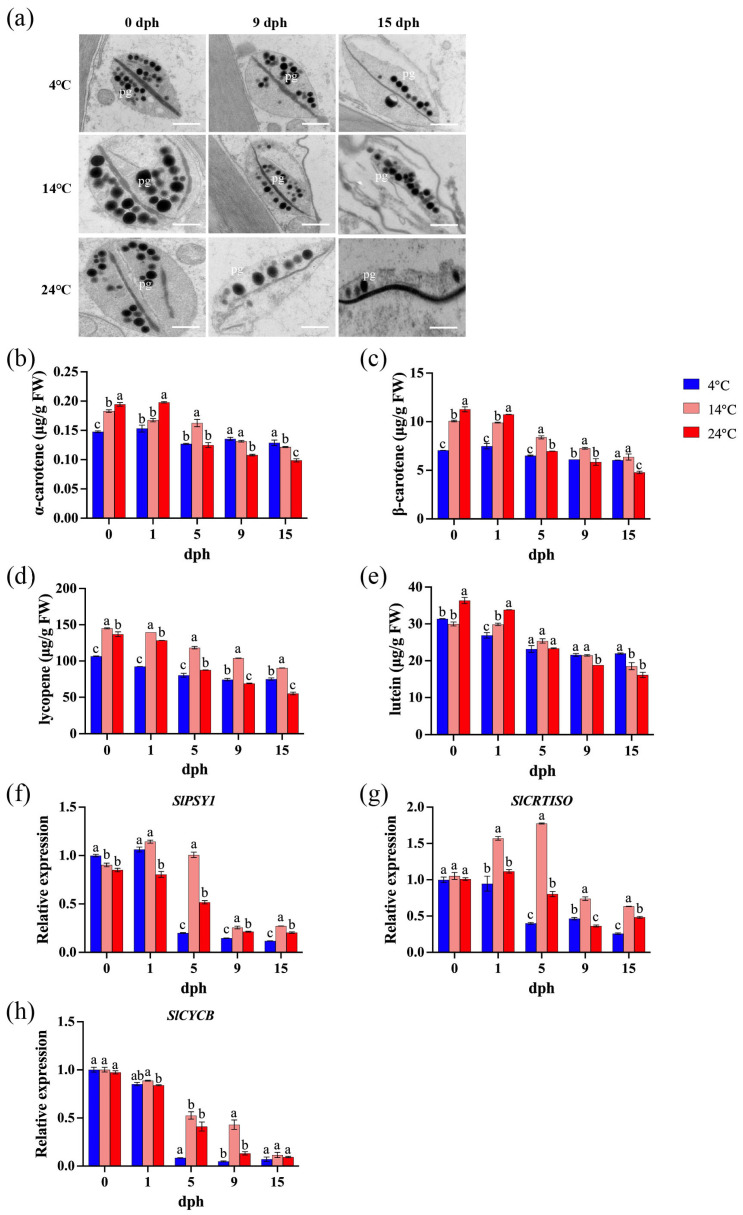
Carotenoid biosynthesis during storage (0, 1, 5, 9, 15 dph) at different temperatures (4, 14, 24 °C). (**a**) Plastid ultrastructure. pg: plastoglobuli. Scale bar, 600 nm; (**b**) α-carotene content; (**c**) β-carotene; (**d**) lycopene; (**e**) lutein. FW, fresh weight. (**f**) Relative expression of *SlPSY1* gene; (**g**) relative expression of *SlCRTISO* gene; (**h**) relative expression of *SlCYCB* gene. dph, days postharvest. The data indicate averages ± standard deviations (SDs). Different lowercase letters indicate statistically significant differences (*p* < 0.05) as determined by a one-way ANOVA test.

Within plant cells, plastids serve as the primary organelles responsible for both the synthesis and storage of carotenoids. Specifically, chromoplasts are plastids characterized by their accumulation of carotenoids. Transmission electron microscopy [45] analysis demonstrated that prolonged storage duration leads to a progressive degradation of plastid ultrastructure, and this degradation process is significantly exacerbated by elevated storage temperatures (Figure 4a). Carotenoid content decreased more rapidly during storage with increasing temperature. After 15 days of storage at 4 °C, the levels of α-carotene, β-carotene, lycopene, and lutein decreased by 12.8%, 14.2%, 12.8%, and 30.0%, respectively. Storage at 14 °C resulted in reductions of 33.5%, 36.8%, 33.5%, and 38.2%, respectively, while storage at 24 °C led to decreases of 49.2%, 57.6%, 49.2%, and 55.5%, respectively. Furthermore, at 15 dph, the carotenoid content in tomatoes stored at 24 °C was significantly lower than that in tomatoes stored at lower temperatures (Figure 4b–e). The expression levels of *SlPSY1*, *SlCRTISO*, and *SlCYCB* generally exhibited a decreasing trend under all storage temperatures, with a particularly sharp decline observed between 1 and 5 dph (Figure 4f–h). An exception was observed in tomatoes stored at 14 °C, where *SlCRTISO* and *SlCYCB* expression initially increased before decreasing sharply before 5 dph (Figure 4g).

### 3.4. Volatile Compounds of Tomato Fruit During Storage

A comprehensive analysis identified 115 volatile compounds, categorized into 22 acids, 20 hydrocarbons, 17 esters, 15 aldehydes, 14 alcohols, 14 ketones, and 13 miscellaneous compounds (including furans, nitriles, and thiazoles) (Table S1). The release of some compounds, like Butanoic acid, Hexanoic acid, 2-methyl-Propanal, 2-pentyl-Furan, Hexanoic acid, 3-Octanone, and Acetaldehyde, increased notably with higher temperature. However, a small portion of compounds, including 3-Butenenitrile, nonanal, and n-Caproic acid vinyl ester, decreased in release with increasing temperature. Acids constituted the predominant group, representing 19.13% of the total volatile compounds detected (Table S1). To elucidate the influence of storage temperature and duration on the volatile profiles of tomatoes, principal component analysis (PCA) was conducted. The first 14 principal components collectively accounted for 100% of the total variance (Table S2). Based on a cumulative variance contribution threshold of ≥85%, seven principal components (PC1–PC7), explaining 87.67% of the variance, were selected (Table S2). These components were primarily characterized by high loadings of hydrocarbons, alcohols, and esters. PC1–PC7 were subsequently employed to represent the overall volatile profile and evaluate tomato flavor characteristics (Table S3). The comprehensive flavor score indicated that freshly harvested tomatoes (0 dph at 4 °C, 14 °C, and 24 °C) exhibited the second-highest flavor intensity, indicating a relatively robust flavor profile immediately after harvest. The highest flavor rating was assigned to tomatoes stored at 4 °C for 5 dph. At a storage temperature of 14 °C, the peak tomato flavor intensity was observed at 9 dph. Conversely, for tomatoes stored at 24 °C, flavor intensity initially declined (0–9 dph) before subsequently increasing (9–15 dph) (Table 1).

**Figure 5:**
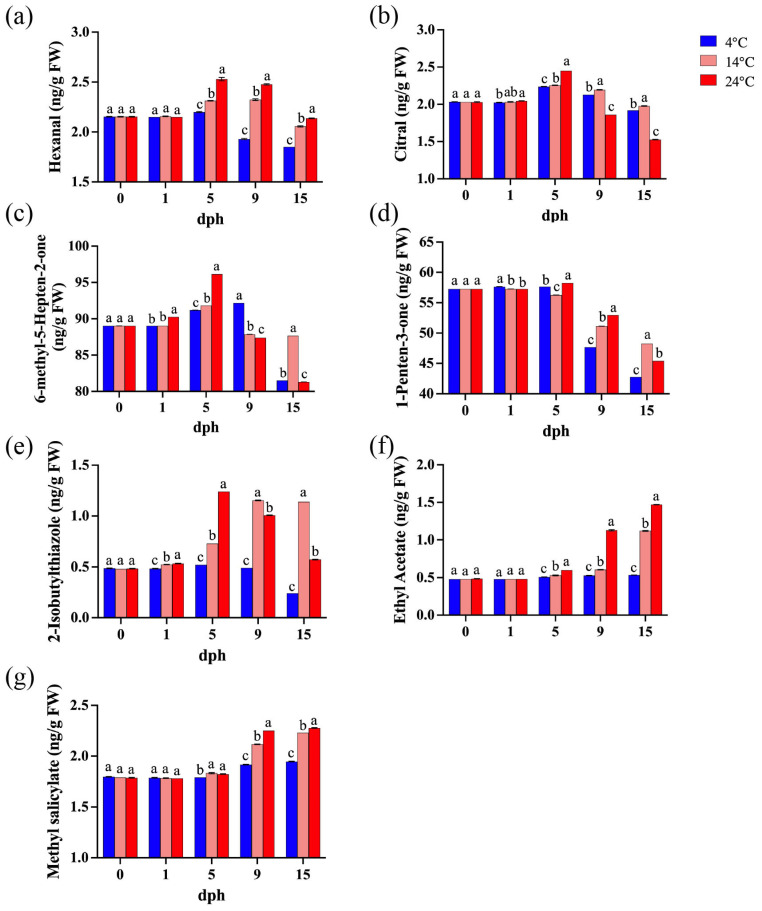
Important volatile compounds during storage (0, 1, 5, 9, 15 dph) at different temperatures (4, 14, 24 °C). (**a**) Hexanal; (**b**) Citral; (**c**) 6-methyl-5-Hepten-2-one; (**d**) 1-Penten-3-one; (**e**) 2-Isobutylthiazole; (**f**) Ethyl acetate; (**g**) Methyl salicylate. dph, days postharvest. FW, fresh weight. The data indicate averages ± standard deviations (SDs). Different lowercase letters indicate statistically significant differences (*p* < 0.05) as determined by a one-way ANOVA test.

Seven volatile compounds closely associated with tomato aroma were identified [28,46], including five known contributors to tomato flavor: hexanal, citral, 6-methyl-5-hepten-2-one, 1-penten-3-one, and 2-isobutylthiazole. The concentrations of hexanal, citral, and 6-methyl-5-hepten-2-one generally increased from 1 to 5 dph, and then gradually decreased with the lowest concentration at 15 dph (Figure 5a–c). Furthermore, the concentration of 1-penten-3-one began to decline from 5 dph (Figure 5d). The concentration of 2-isobutylthiazole in tomatoes stored at 14 °C and 24 °C increased significantly from 5 dph, whereas it decreased markedly between 9 and 15 dph at 4 °C. (Figure 5e). Conversely, two volatile compounds associated with undesirable tomato flavor (ethyl acetate and methyl salicylate) were also detected. Their concentrations remained relatively stable until 5 dph, after which they showed increasing trends (Figure 5f,g). In addition, after 5 dph, the concentrations of most volatile compounds in tomatoes stored at higher temperatures (14 °C and 24 °C) were significantly higher than those stored at 4 °C, with the exception of citral (Figure 5). Therefore, higher storage temperatures promoted the release of aroma compounds after 5 dph, but also enhanced the emission of undesirable volatile compounds.

## 4. Discussion

Consumers prioritize fruit color and juice content when selecting tomatoes [47], with a preference for those exhibiting a rich aroma and moderate softening [39,46]. Storage temperature is a critical factor influencing the postharvest quality of fruits [38]. This study investigated the effects of varying storage temperatures on the postharvest quality attributes of mature tomatoes. Tomatoes were maintained at 4 °C, 14 °C, and 24 °C, and their external appearance, firmness, carotenoid metabolism, and flavor profiles were evaluated at 0, 1, 5, 9, and 15 dph. The findings offer a theoretical foundation for optimizing tomato marketing strategies, informing consumer storage practices, and minimizing postharvest losses.

Total soluble solids (TSS) content is a primary indicator of fruit maturity and quality. Tomatoes stored at 4 °C and 14 °C exhibited the highest TSS content at 5 dph, indicating optimal eating quality. At these temperatures, the TSS content initially increased and then decreased, potentially due to the conversion of starch into soluble sugars (Figure 1b). At 15 dph, the TSS content of tomatoes stored at 4 °C and 14 °C was significantly higher than that of tomatoes stored at 24 °C (Figure 1b), likely due to the suppression of respiration at lower temperatures [48]. This suggests that low temperatures play a role in maintaining TSS levels in tomatoes. Storage temperatures of 5 °C and 10 °C had less impact on tomato cv. Belle [49], similar to the findings of this study, implying that TSS loss is more pronounced at higher storage temperatures. Furthermore, storage at 4 °C was beneficial in reducing ascorbic acid (AsA) loss, with AsA content significantly higher at 9–15 dph compared to storage at 14 °C and 24 °C (Figure 1c). However, AsA content gradually decreased during storage (Figure 1c), which is attributed to its antioxidant function [50,51].

Storage at 4 °C and 14 °C had similar effects on delaying postharvest softening of tomatoes (Figure 2a), and both temperatures were more effective than storage at 24 °C in reducing water loss (Figure 2b). The previous study found that lower storage temperature (5 °C) delayed fruit softening and reduced weight loss of tomato cv. Belle [49]. Moreover, increasing the temperature from 18–20 °C to 26 °C shortened the shelf life of all 41 tomato genotypes by approximately 4 days and increased their susceptibility to diseases [52]. Studies have shown that changes in blueberry firmness are related to water loss, with a strong correlation between fruit weight loss and firmness, indicating that reduced firmness is a result of dehydration [53]. Additionally, low-temperature storage had a more pronounced effect on maintaining the structure of fruit pericarp cells, potentially due to the influence of temperature on the transcriptional expression of primary cell wall genes, like *SlCESA6*, *SlEXP1*, and *SlPL* (Figure 2d–g). This conclusion was supported by enzyme activity assays, which revealed that the activities of PG, β-Gal, and cellulase were significantly higher in tomatoes stored at 24 °C compared to those stored at 4 °C and 14 °C (Figure 3a,b). Previous research has shown that low-temperature storage reduces PG activity [54], which is consistent with the results of this study (Figure 3a); however, β-Gal activity increased, and cellulase activity remained unchanged at low temperatures [54]. Recent studies have indicated that *SlPL*, *SlCEL2*, and *SlEXP1* are involved in the softening process of tomato fruit after ripening [39,55,56]. Low-temperature (4 °C) storage significantly reduced the transcriptional levels of cell wall softening-related genes (*SlPL*, *SlCEL2*, *SlEXP1*) [57], which generally aligns with the findings of this study (Figure 2e–g). In conclusion, low-temperature storage prolongs fruit shelf life by inhibiting fruit softening. This inhibition is achieved through the reduced activity of cell wall-degrading enzymes and the suppression of gene transcription.

Carotenoids are major nutritional components of mature tomato fruits and key pigments determining fruit color, which is an important commercial attribute [58]. In this study, the ultrastructure of chromoplasts in tomatoes stored at low temperatures (4 °C and 14 °C) was relatively intact (Figure 4a). Moreover, the levels of α-carotene, β-carotene, lycopene, and lutein were more stable and degraded more slowly in tomatoes stored at 4 °C compared to those stored at 14 °C and 24 °C (Figure 4b,c). Furthermore, the transcription levels of three carotenoid biosynthesis-related genes (*SlPSY1*, *SlCRTISO*, *SlCYCB*) were influenced by low temperature, primarily being suppressed during the 1–9 dph stage (Figure 4f,g). This is consistent with other studies showing that carotenoid synthesis genes are suppressed by low temperatures [30,59], demonstrating that low-temperature storage can better maintain carotenoid content in tomatoes, keeping them fresh for a longer period. However, in grapefruits stored at 2 °C, the levels of 9-Z-violaxanthin (yellow) and β-citraurin (orange-yellow) were significantly lower than those stored at 12 °C [20].

Volatile compounds are important components of tomato flavor [60], derived from fatty acids, carotenoids, phenylalanine, and branched-chain amino acids [61]. Among these, only 16 compounds are considered to contribute to tomato flavor [28,62]. This study detected seven of these compounds, with five contributing to the characteristic tomato flavor: 6-methyl-5-hepten-2-one (fruity, floral), 1-penten-3-one (sweet, fruity, grassy), hexanal (green, grassy), citral (fresh), and 2-isobutylthiazole (green, tomato vine) (Figure 5a–e). Two compounds that impair tomato flavor were also identified: methyl salicylate (wintergreen) and ethyl acetate (Figure 5f,g). Together, these compounds determine the fresh flavor of tomatoes. Low temperature significantly inhibited the expression of genes related to the biosynthesis of volatile compounds in stored tomatoes [63], influencing the composition and concentration of volatile compounds responsible for fruit aroma [64]. In this study, the levels of 2-isobutylthiazole, hexanal, citral, and 1-penten-3-one reached their maximum at 5 dph under different temperature treatments. Based on the comprehensive score, tomatoes stored at low temperatures had better flavor at 5 dph, while the flavor deteriorated during later storage periods due to lower concentrations of volatile compounds (Table 1). In addition, lower levels of alcohols, esters, and hydrocarbons were detected (Table S1). However, the levels of two undesirable volatile compounds, methyl salicylate and ethyl acetate, gradually increased during storage, with notable higher levels at 14 °C and 24 °C compared to 4 °C (Figure 5f,g). Low temperature not only resulted in a milder tomato flavor, but also suppressed the production of undesirable volatile compounds. Therefore, this study supports the conclusion that tomato quality initially improves and then declines with increasing storage time. While the flavor of tomatoes stored at low temperatures may be less intense, the production of detrimental volatile compounds is also inhibited.

This study found that 4 °C and 14 °C temperature storage had generally similar effects on maintaining the postharvest quality of tomatoes, both delaying nutrient loss, fruit softening, and carotenoid degradation. In conclusion, considering both postharvest tomato quality and storage cost-effectiveness, a temperature of 14 °C is the preferred storage condition, providing a basis for low-temperature transportation or storage of tomatoes after harvest. Storage at 14 °C achieves comparable quality preservation to storage at 4 °C, while simultaneously encouraging the desirable release of volatile aroma compounds. Furthermore, tomatoes stored at 14 °C are best consumed within five days. During this period, they exhibit high soluble solid and sucrose content and optimal release of desirable aromatic compounds. Beyond five days, fruit softening accelerates, ascorbic acid degradation increases, and the production of undesirable volatile compounds is accelerated.

## Figures and Tables

**Table 1:** Principal component score and comprehensive score.

	PC1	PC2	PC3	PC4	PC5	PC6	PC7	Score	Rank
4–5	1.37	0.52	0.20	0.35	0.18	0.03	0.07	2.71	1
4–0	0.93	0.35	0.13	0.23	0.12	0.02	0.05	1.83	2
24–0	0.93	0.35	0.13	0.23	0.12	0.02	0.05	1.83	2
14–0	0.93	0.35	0.13	0.23	0.12	0.02	0.05	1.83	2
24–1	0.41	0.16	0.06	0.10	0.05	0.01	0.02	0.82	3
24–15	0.35	0.13	0.05	0.09	0.05	0.01	0.02	0.69	3
14–9	0.19	0.07	0.03	0.05	0.02	0.00	0.01	0.38	4
14–5	0.15	0.06	0.02	0.04	0.02	0.00	0.01	0.31	5
24–5	0.10	0.04	0.01	0.03	0.01	0.00	0.01	0.20	6
4–9	−0.10	−0.04	−0.01	−0.03	−0.01	0.00	−0.01	−0.20	7
4–1	−0.35	−0.13	−0.05	−0.09	−0.05	−0.01	−0.02	−0.69	8
24–9	−0.35	−0.13	−0.05	−0.09	−0.05	−0.01	−0.02	−0.69	9
4–15	−0.38	−0.14	−0.06	−0.10	−0.05	−0.01	−0.02	−0.76	10
14–1	−0.53	−0.20	−0.08	−0.13	−0.07	−0.01	−0.03	−1.05	11
14–15	−3.64	−1.37	−0.53	−0.92	−0.47	−0.09	−0.18	−7.20	12

PC, principal component; 4–0, 4–1, 4–5, 4–9, 4–15 indicate 4 °C treatment for 0, 1, 5, 9, 15 days postharvest (dph), respectively; 14–0, 14–1, 14–5, 14–9, 14–15 indicate 14 °C treatment for 0, 1, 5, 9, 15 dph, respectively; 24–0, 24–1, 24–5, 24–9, 24–15 indicate 24 °C treatment for 0, 1, 5, 9, 15 dph, respectively.

## Data Availability

The original contributions presented in the study are included in the article/Supplementary Material, further inquiries can be directed to the corresponding author.

## Supplementary Materials

The following supporting information can be downloaded at: https://www.mdpi.com/article/10.3390/foods14061002/s1, Table S1: 115 volatile substances were detected in tomato fruit (ng/g FW). Table S2: Characteristic value and variance contribution rates of principal components. Table S3: Principal component loading diagram of each volatile substance of tomato. Table S4: The primers used in this study.
