# Supplementary material for: Effect of Postharvest Storage Temperature and Duration on Tomato Fruit Quality
Source: Foods. 2025 Mar 15;14(6):1002. doi: 10.3390/foods14061002 (PMC11941398; doi:10.3390/foods14061002)
Supplement: Supplementary file 1 [file foods-14-01002-s001.zip › foods-3526161-supplementary.pdf]

Supplemental Table S1 115 volatile substances were detected in tomato fruit (ng/g FW)

number	volatile substances	4-0	4-1	4-5	4-9	4-15	14-0	14-1	14-5	14-9	14-15	24-0	24-1	24-5	24-9	24-15
X1	Hexamethylcyclotrisiloxane	145.46	124.92	152.61	128.89	124.33	145.46	121.95	133.03	133.63	71.88	145.46	137.19	132.18	124.87	136.12
X2	Ethyl Acetate	0.48	0.48	0.51	0.53	0.53	0.48	0.48	0.53	0.61	1.12	0.48	0.48	0.60	1.13	1.47
X3	Methyl Alcohol	32.48	32.47	32.06	25.26	34.83	32.47	32.47	32.28	27.02	22.52	32.47	32.47	24.57	29.17	20.16
X4	Ethanol	1536.99	1536.98	2380.24	1648.95	1524.65	1536.99	1536.99	1723.27	1760.36	1735.84	1536.99	1536.98	1529.09	1547.92	1604.33
X5	Octamethylcyclotetrasiloxane	70.42	70.42	68.63	60.63	61.01	70.41	70.41	68.12	71.43	87.04	70.42	70.41	70.52	71.96	74.07
X6	Hexanal	2.15	2.15	2.20	1.93	1.85	2.15	2.16	2.32	2.32	2.06	2.16	2.15	2.53	2.48	2.14
X7	dodecamethyl-Cyclohexasiloxane	8.81	8.81	9.67	10.24	7.43	8.81	8.81	8.24	9.56	12.63	8.81	8.81	10.57	9.44	9.25
X8	2-Isobutylthiazole	0.48	0.48	0.52	0.49	0.24	0.48	0.52	0.73	1.16	1.14	0.48	0.53	1.24	1.01	0.57
X9	tetradecamethyl-Cycloheptasiloxane	3.40	3.42	3.64	2.92	3.13	3.41	3.41	3.16	3.52	4.12	3.41	3.41	3.30	3.39	3.37
X10	Propanoic acid	0.40	0.41	1.22	0.84	1.43	0.41	0.41	0.61	0.54	0.31	0.41	0.41	0.68	0.53	0.54
X11	3-bromo-Pentane	0.24	0.25	0.18	0.29	0.13	0.25	0.24	0.31	0.82	1.04	0.24	0.26	0.27	0.27	0.47
X12	4-acetyl-2,3,4,5,5-pentamethyl-2-Cyclopenten-1-one	0.20	0.22	0.26	0.33	0.22	0.21	0.21	0.31	0.33	0.47	0.21	0.21	0.33	0.53	0.88
X13	3,4-Dihydroxyphenylglycol, 4TMS derivative	2.18	2.18	2.24	1.72	1.70	2.02	2.18	2.29	2.19	2.19	2.18	2.18	2.18	2.68	2.79
X14	Butanoic acid	0.67	0.67	1.29	0.88	0.51	0.67	0.67	1.27	1.42	1.48	0.68	0.68	1.57	2.04	2.41
X15	Benzoic acid, 2-hydroxy-, ethyl ester	213.49	213.47	198.14	142.27	130.81	213.49	213.48	191.63	168.72	152.30	213.49	213.48	182.84	167.33	126.68
X16	Hexanoic acid	9.55	9.55	12.51	9.47	6.95	9.55	9.55	11.05	15.02	16.37	9.56	9.55	18.27	24.64	25.49
X17	l2-methoxy-Pheno	82.47	82.47	40.95	33.34	26.75	82.47	82.47	107.06	110.98	115.29	82.47	82.47	66.04	52.74	41.41
X18	Propanoic acid, 2-methyl-, anhydride	0.17	0.17	0.25	0.29	0.31	0.16	0.17	0.23	0.52	0.48	0.17	0.17	0.18	0.31	0.35
X19	Tributyl phosphate	0.47	0.47	1.13	1.25	1.41	0.47	0.47	1.16	0.93	0.64	0.47	0.47	1.05	0.91	0.69
X20	Terephthalic acid, tridec-2-yn-1-yl ethyl ester	0.32	0.32	0.35	0.34	0.29	0.31	0.31	0.35	0.41	0.34	0.31	0.32	0.46	0.36	0.28
X21	4-Fluorobenzoic acid, tridec-2-ynyl ester	0.14	0.13	0.24	0.25	0.19	0.14	0.13	0.19	0.24	0.20	0.14	0.13	0.17	0.27	0.39
X22	1,1,1,5,7,7,7-Heptamethyl-3,3-bis (trimethylsiloxy)tetrasiloxane	0.69	0.68	0.65	0.53	0.51	0.69	0.68	0.52	0.60	0.61	0.69	0.68	0.52	0.43	0.48
X23	Hexadecanoic acid, ethyl ester	0.41	0.41	0.43	0.55	0.46	0.41	0.40	0.78	1.61	1.34	0.42	0.42	0.89	1.79	1.25

X24	2-methyl-Propanal	0.49	0.48	0.31	0.14	0.14	0.48	0.48	0.53	0.62	0.99	0.49	0.49	0.71	1.09	1.34
X25	3,3-dimethyl-Hexane	2.92	2.92	3.53	4.16	3.17	2.92	2.92	3.68	4.31	3.53	2.93	2.93	4.31	4.06	3.12
X26	6-methyl-5-Hepten-2-one	89.03	89.02	91.18	92.17	81.53	89.02	89.02	91.82	87.87	87.67	89.03	90.25	96.14	87.38	81.30
X27	1-Hexanol	3.67	3.68	6.52	8.87	9.98	3.67	3.68	7.27	10.11	13.54	3.67	3.67	7.44	11.22	15.46
X28	3-(4-methyl-3-pentenyl)-Furan	2.18	2.17	2.87	3.77	2.97	2.17	2.17	2.89	3.76	3.87	2.17	2.17	3.12	3.43	4.79
X29	Ammonium acetate	1.48	1.47	1.97	1.89	1.32	1.47	1.47	2.56	2.58	3.31	1.48	1.48	2.43	2.20	1.74
X30	Dodecanedioic acid, 2TBDMS derivative	0.26	0.26	0.22	0.17	0.16	0.25	0.26	0.23	0.19	0.15	0.26	0.26	0.27	0.26	0.2
X31	Acetic acid	0.29	0.28	0.25	0.21	0.15	0.28	0.28	0.27	0.26	0.20	0.28	0.28	0.22	0.19	0.14
X32	Tetrahydropyran Z-10-dodecenoate	1.43	1.42	1.62	1.13	0.89	1.43	1.42	1.95	1.71	1.63	1.42	1.42	1.96	1.59	1.26
X33	2-hexyl-1,3-Dioxolane	0.28	0.28	0.31	0.16	0.15	0.28	0.28	0.35	0.25	0.15	0.28	0.28	0.39	0.25	0.15
X34	Phthalic acid, cyclobutyl tridecyl ester	0.24	0.24	0.25	0.21	0.17	0.24	0.24	0.36	0.23	0.16	0.24	0.24	0.43	0.21	0.15
X35	decamethyl-Cyclopentasiloxane	21.61	21.63	27.64	20.14	17.92	21.62	21.63	27.93	20.69	13.93	21.62	21.62	30.13	35.41	25.46
X36	2-pentyl-Furan	45.81	45.81	49.44	75.21	58.54	45.82	45.83	56.49	76.93	81.04	45.82	45.82	57.84	87.27	116.66
X37	Hexanoic acid, ethyl ester	2.31	2.34	3.27	4.16	5.96	2.33	2.33	3.33	5.14	8.15	2.32	2.32	3.77	9.09	9.88
X38	3-Octanone	2.58	2.58	4.25	4.65	4.75	2.58	2.57	4.34	5.27	7.33	2.59	2.58	5.04	5.54	8.100
X39	2-Ethylhexanal ethylene glycol acetal	0.50	0.51	0.66	1.19	1.57	0.50	0.52	0.96	2.11	4.16	0.51	0.52	0.99	2.35	4.41
X40	3,7-dimethyl-, (E)-2,6-Octadienal	6.32	4.67	4.38	2.89	2.25	6.33	5.42	5.48	3.32	3.31	6.33	6.03	5.93	3.69	1.92
X41	1,1,1,3,5,7,7,7-Octamethyl-3,5-bis (trimethylsiloxy)tetrasiloxane	0.41	0.42	0.30	0.24	0.21	0.42	0.23	0.22	0.26	0.29	0.41	0.29	0.21	0.23	0.21
X42	Acetaldehyde	1.36	1.35	1.66	2.05	1.26	1.36	1.27	1.45	3.02	3.07	1.34	1.32	2.42	3.59	4.08
X43	3,4-dihydroxy-3,4-dimethyl-2,5-Hexanedione	1.14	1.02	1.15	1.02	1.01	1.13	1.13	0.49	0.48	0.27	1.13	0.84	0.52	0.45	0.71
X44	(E,Z)-2,4-Decadienal	1.36	1.36	1.69	1.48	1.34	1.37	1.36	2.12	2.91	1.51	1.35	0.23	0.66	0.38	0.16
X45	Methyl salicylate	1.8	1.78	1.79	1.91	1.95	1.79	1.79	1.83	2.12	2.23	1.79	1.78	1.82	2.25	2.28
X46	Methyl vinyl ketone	2.14	2.76	2.86	1.05	0.98	2.16	3.12	3.52	2.87	2.67	2.15	2.96	3.87	2.63	2.85
X47	3-Butenenitrile	7.74	6.88	9.04	10.56	6.15	7.72	5.78	9.62	9.84	5.07	7.67	3.18	5.00	6.06	3.65
X48	2-hydroxy-3,3-dimethyl-Butanoic acid	0.33	0.23	0.71	0.69	0.54	0.32	0.33	0.37	0.67	0.31	7.78	0.45	0.84	0.92	1.38

X49	Citral	2.03	2.02	2.23	2.13	1.92	2.03	2.03	2.25	2.19	1.98	2.03	2.05	2.45	1.86	1.52
X50	1,3-Dioxolane-2-methanol	0.09	0.10	0.14	0.09	0.10	0.10	0.13	0.15	0.16	0.19	0.09	0.12	0.12	0.09	0.09
X51	Nonanal	0.68	0.66	0.78	0.91	0.52	0.69	0.70	0.76	0.76	0.47	0.68	0.15	0.52	0.61	0.21
X52	1-Pentanol	-	9.16	14.72	14.72	11.72	-	9.20	14.67	15.79	-	-	9.18	15.44	16.37	13.78
X53	O-(3-methylbutyl)-Hydroxylamine	1.34	2.97	3.46	0.88	-	0.91	5.70	9.11	3.56	7.06	0.64	3.61	2.58	0.30	0.38
X54	Octanoic acid, ethyl ester	0.38	0.26	0.28	0.40	0.48	0.38	0.50	0.37	0.49	0.95	0.37	0.44	0.64	1.26	1.55
X55	2,2,5-trimethyl-3,4-Hexanedione	1.85	2.44	2.53	2.22	2.23	1.84	1.96	1.65	-	1.46	2.47	1.95	1.40	-	-
X56	(Z)-Decanoic acid, 3-hexenyl ester	0.15	0.30	0.43	0.40	0.34	0.14	0.26	0.31	0.37	-	0.15	0.27	-	0.47	-
X57	Acetic anhydride	-	-	-	0.01	0.05	-	0.09	0.10	0.66	0.51	-	0.07	0.07	0.19	0.20
X58	Toluene	0.18	0.23	0.33	-	0.23	0.18	0.27	-	0.38	0.30	0.17	0.27	0.28	0.22	0.32
X59	n-Caproic acid vinyl ester	8.36	11.27	12.56	10.34	10.36	8.36	8.34	-	-	0.63	8.35	5.68	3.44	-	0.69
X60	(E)-4-(3-((tert-Butyldimethylsilyl)oxy)-3-oxoprop-1-en-1-yl)-1,2-phenylene bis(2,2,2-trifluoroacetate)	-	-	-	2.70	2.50	-	5.99	9.83	10.56	13.53	-	4.54	5.73	4.37	4.30
X61	1-Penten-3-one	57.26	57.64	57.65	47.68	42.75	57.25	57.26	56.26	51.13	48.26	57.26	57.26	58.26	52.95	45.41
X62	Benzaldehyde	0.24	0.25	0.36	0.27	0.14	0.25	0.25	0.26	0.54	0.61	0.25	0.28	0.24	0.09	0.13
X63	trans-2-Nonenal	0.51	0.37	0.47	0.56	0.59	0.50	0.55	0.56	0.90	1.13	0.50	-	-	-	-
X64	3-methoxy-3-methyl-2-Butanone	-	-	0.40	0.42	0.79	-	0.66	1.11	1.28	1.94	-	-	-	-	-
X65	Cyclobutanol	0.11	0.08	0.06	0.08	-	0.11	0.42	0.30	-	-	0.11	0.13	0.54	0.48	0.23
X66	di-tert-Butyl dicarbonate	0.38	0.26	0.44	0.31	0.4	0.38	0.74	0.77	0.85	0.6	0.38	-	-	-	-
X67	3-Pentanone	-	-	15.52	12.65	10.67	-	14.29	12.73	12.69	11.47	-	15.38	13.47	13.39	12.05
X68	4-methyl-1-(1-methylethyl)-bicyclo[3.1.0]hex-2-ene	-	5.28	6.71	5.74	6.34	-	7.72	9.40	10.60	13.85	-	7.85	7.93	10.12	11.69
X69	Acetone	-	-	-	0.74	0.77	-	0.52	0.54	0.71	0.91	-	0.55	1.02	2.73	3.45
X70	(E)-2-Heptenal	-	-	0.92	1.32	1.31	-	0.90	0.74	0.48	0.37	-	-	0.73	0.69	0.70
X71	o-Allylhydroxylamine	-	-	0.22	0.39	0.52	-	0.19	0.21	-	-	-	-	0.41	0.61	0.77
X72	1-vinyl-1H-Pyrazole	0.12	0.12	0.11	0.16	0.14	0.13	0.14	-	0.15	0.15	0.13	0.03	-	-	-

X73	Hydrazinecarboxamide	-	-	43.82	47.43	51.33	21.42	21.43	26.96	-	-	21.42	21.43	27.65	32.95	-
X74	Propanoic acid, 2-hydroxy-, ethyl ester	-	-	-	0.67	0.82	-	0.71	0.9	1.17	1.29	-	4.85	5.09	5.86	10.92
X75	2-ethyl-1-Hexanol	-	0.39	0.85	1.06	0.87	-	0.22	0.33	0.28	0.79	-	0.23	-	-	-
X76	Propyl pyruvate	0.07	0.06	-	-	-	0.08	-	-	1.51	1.23	-	0.18	0.18	0.24	0.49
X77	4-hydroxy-Benzaldehyde	23.19	23.18	21.81	21.11	20.87	23.19	23.18	-	-	-	23.18	23.19	-	-	-
X78	4-Penten-1-ol, propanoate	-	-	4.59	-	-	2.53	3.35	2.65	2.45	-	2.53	4.89	5.82	3.43	-
X79	Pyruvic acid, butyl ester	0.35	0.35	0.11	0.13	0.05	0.34	0.26	0.27	0.20	-	0.34	0.11	0.22	-	-
X80	methyl-Benzenemethanol	-	4.26	-	-	-	2.83	6.19	4.46	4.64	1.40	-	8.86	9.54	12.38	12.19
X81	1-(2-furanyl)-3-Butene-1,2-diol	-	-	-	-	0.26	0.23	0.33	0.50	0.95	0.67	0.25	0.31	-	-	0.17
X82	Phosphoric acid, dimethyl pentyl ester	-	-	0.07	0.05	0.06	0.03	0.04	0.08	0.04	0.04	-	0.09	0.08	-	0.05
X83	2-Hexenal	6.11	6.11	9.57	11.62	10.60	6.11	-	-	-	-	6.11	12.17	8.35	-	-
X84	2-Hexenoic acid, ethyl ester	-	-	-	0.43	0.71	0.53	0.53	-	1.03	1.93	0.25	0.34	-	0.45	0.48
X85	Pentanoic acid, ethyl ester	-	-	-	-	0.68	-	0.61	0.34	1.13	0.82	-	0.38	-	0.4	0.49
X86	2-nitro-Propane	0.02	-	-	-	-	0.02	0.06	0.08	-	-	0.02	0.06	0.02	0.05	-
X87	1-(3-hydroxyphenyl)-Ethanone	-	0.05	0.04	0.07	0.06	-	0.21	0.16	0.17	-	-	0.15	0.10	-	-
X88	3-methyl-1-Butanol	0.46	0.35	-	-	-	-	2.43	1.43	3.57	9.08	-	0.93	0.43	0.29	2.14
X89	1-Heptanol	-	-	-	1.43	0.70	0.46	0.79	-	-	2.49	-	-	1.18	1.87	1.78
X90	2-hexyl-Furan	1.14	1.03	0.80	0.70	0.59	1.03	1.15	-	0.95	-	1.03	-	-	-	-
X91	Pentanal	-	8.32	9.67	10.41	-	-	-	-	-	-	-	-	0.33	-	-
X92	4-Methyl-2,4-bis(p-hydroxyphenyl)pent-1-ene, 2TMS derivative	-	0.09	0.03	0.09	0.12	0.04	-	-	0.05	-	0.05	0.18	0.18	0.13	0.15
X93	5-Ethylcyclopent-1-enecarboxaldehyde	-	0.60	0.37	-	0.79	-	-	-	-	-	-	0.28	-	0.11	-
X94	2-(2-furyl)(2-pyrimidylamino)methylphenol	0.64	0.66	3.3	-	-	-	-	2.54	6.47	3.40	0.51	-	-	-	-
X95	2-(3-methyl-2-cyclopenten-1-yl)-2-methylpropionaldehyde	-	0.10	0.11	0.12	-	0.29	-	0.12	0.22	-	0.22	-	-	-	-
X96	Vinyl crotonate	-	0.12	0.10	0.09	0.11	0.03	0.09	-	0.01	-	-	-	-	-	-
X97	2-Methyl-2-propyl methylphosphonofluoridate	-	-	-	-	-	-	0.46	0.38	0.16	-	-	0.41	-	0.12	0.18

X98	Stearic acid, TBDMS derivative	-	-	0.42	0.37	-	-	-	0.42	-	-	0.47	-	-	0.36	0.40
X99	2-Octanol	-	-	17.66	15.4	-	20.92	-	-	-	-	-	-	4.61	-	-
X100	1,2-diethoxy-Ethane	0.13	0.16	0.25	0.74	-	-	-	0.37	-	-	0.13	0.23	0.26	-	-
X101	Diazenecarboximidoyl bromide	-	0.49	0.53	-	0.38	-	-	-	-	-	0.48	0.23	-	-	-
X102	4-Heptanol	-	-	-	-	0.05	0.05	0.06	0.06	0.08	0.20	-	0.16	-	-	-
X103	2-hexyl-1,3-Dioxolane	0.07	-	0.05	-	0.05	0.07	-	-	-	-	0.07	-	0.06	0.07	-
X104	4-methyl-1-Penten-3-one	-	-	-	-	0.10	-	0.05	0.05	0.35	0.48	-	-	-	0.06	0.27
X105	1-nitro-Propane	-	-	0.05	0.04	-	-	0.04	0.08	0.23	0.31	-	-	-	-	-
X106	4,5-dimethyl-2,6-Octadiene	-	-	-	-	-	-	-	2.64	2.65	1.94	-	-	-	0.28	0.32
X107	(S)-Isopropyl lactate	-	-	-	0.28	-	-	0.55	0.85	0.64	0.46	-	-	-	-	0.54
X108	3,4-dimethyl-1,5-Heptadiene	-	-	-	-	-	-	-	0.88	1.02	1.45	-	0.08	0.45	0.19	0.17
X109	6-methyl-2-propyl-4(1H)-Pyrimidinone	-	-	-	-	0.15	0.15	0.15	0.15	0.12	0.26	-	-	-	0.08	0.18
X110	3-ethyl-2-methyl-1-Pentene	0.48	0.47	0.54	0.23	-	0.47	-	-	-	-	0.47	-	-	-	-
X111	Di-tert-butyl peroxide	-	-	-	-	-	-	0.07	0.2	1.33	0.09	-	-	0.34	0.47	0.45
X112	2-Methoxy-4-vinylphenol	-	-	-	-	-	-	0.63	0.21	0.93	1.27	-	-	-	0.37	0.44
X113	6-ethyl-2-methyl-Decane	-	-	-	-	-	0.63	0.54	0.82	1.19	0.53	-	-	-	0.43	-
X114	Acetoin	-	-	0.06	0.03	-	-	0.19	0.24	-	0.53	-	-	-	-	0.31
X115	Benzyl alcohol	4.63	5.93	5.92	-	-	4.63	-	-	-	-	4.63	-	-	-	-

The data were the mean values of three biological repeats, and “-” means that the substance was not detected. FW, fresh weight. 4-0, 4-1, 4-5, 4-9, 4-15 indicate 4°C treatment for 0, 1, 5, 9, 15 days post-harvest (dph), respectively; 14-0, 14-1, 14-5, 14-9, 14-15 indicate 14°C treatment for 0, 1, 5, 9, 15 dph, respectively; 24-0, 24-1, 24-5, 24-9, 24-15 indicate 24°C treatment for 0, 1, 5, 9, 15 dph, respectively.

Supplemental Table S2 Characteristic value and variance contribution rates of principal components

principal component	Characteristic value	Variance contribution rates（%）	Cumulative variance contribution rate（%）
1	38.313	33.316	33.316
2	19.578	17.024	50.340
3	14.907	12.962	63.303
4	11.422	9.932	73.234
5	7.124	6.195	79.429
6	5.355	4.656	84.086
7	4.122	3.584	87.670
8	3.585	3.118	90.788
9	2.567	2.232	93.020
10	2.308	2.007	95.027
11	1.880	1.635	96.662
12	1.675	1.456	98.118
13	1.362	1.184	99.302

Supplemental Table S3 Principal component loading diagram of each volatile substance of tomato

volatile substances	PC1	PC2	PC3	PC4	PC5	PC6	PC7
X1	-0.61	-0.32	0.23	0.16	0.26	-0.35	0.20
X2	0.83	-0.40	-0.03	-0.32	0.11	0.03	-0.09
X3	-0.77	0.18	0.01	0.05	-0.27	-0.27	0.22
X4	0.07	0.21	-0.18	0.41	0.38	0.23	0.23
X5	0.58	0.36	0.27	-0.55	0.09	0.33	-0.02
X6	0.21	-0.07	0.88	0.20	0.30	-0.03	0.17
X7	0.55	0.26	0.01	-0.04	0.33	0.64	-0.18
X8	0.68	0.24	0.48	0.19	0.27	0.22	0.09
X9	0.39	0.49	0.07	-0.47	0.15	0.40	0.25
X10	-0.13	-0.31	-0.55	0.60	-0.01	0.07	0.27
X11	0.78	0.53	-0.09	-0.19	0.13	0.11	0.01
X12	0.79	-0.49	0.00	-0.18	0.15	-0.06	-0.13
X13	0.48	-0.34	0.54	-0.31	0.17	-0.17	0.08
X14	0.80	-0.39	0.26	0.06	0.31	0.00	0.00
X15	-0.69	0.36	0.55	-0.20	-0.01	0.01	0.07
X16	0.75	-0.46	0.31	-0.10	0.30	0.02	0.06
X17	0.17	0.81	0.45	-0.23	-0.06	-0.08	-0.08
X18	0.83	0.24	-0.37	0.10	0.18	-0.10	0.20
X19	0.20	-0.22	-0.35	0.83	0.07	0.01	0.02
X20	0.22	0.20	0.47	0.55	0.34	0.23	0.20
X21	0.67	-0.52	-0.22	0.16	0.28	-0.16	-0.01
X22	-0.60	0.58	0.05	-0.41	-0.03	0.13	0.16
X23	0.88	-0.07	0.18	0.04	0.27	-0.16	0.14
X24	0.75	-0.27	0.38	-0.43	0.13	-0.01	-0.04
X25	0.49	-0.02	0.11	0.70	0.38	0.08	0.01
X26	-0.40	0.28	0.52	0.38	0.25	0.38	-0.25
X27	0.90	-0.27	-0.31	0.07	0.11	0.01	-0.03
X28	0.85	-0.27	-0.26	0.16	0.22	0.00	-0.09
X29	0.74	0.40	0.17	0.28	0.23	0.28	-0.14
X30	-0.52	-0.15	0.79	-0.20	0.05	-0.05	0.05
X31	-0.64	0.57	0.43	-0.08	0.00	-0.12	-0.03
X32	0.24	0.36	0.77	0.29	0.25	0.15	-0.03
X33	-0.45	0.18	0.79	0.28	0.10	0.07	0.06
X34	-0.30	0.09	0.71	0.49	0.04	0.16	-0.10
X35	0.04	-0.56	0.62	0.35	0.27	-0.12	0.06
X36	0.85	-0.40	-0.17	-0.05	0.18	-0.13	-0.10
X37	0.86	-0.40	-0.22	-0.12	0.09	-0.03	0.02
X38	0.91	-0.24	-0.20	0.07	0.15	0.12	-0.06
X39	0.92	-0.16	-0.23	-0.24	0.07	0.05	-0.06
X40	-0.63	0.29	0.66	-0.15	-0.02	0.11	-0.12

X41	-0.61	0.32	0.02	-0.59	0.37	0.02	0.03
X42	0.88	-0.31	0.06	-0.09	0.31	-0.02	0.05
X43	-0.85	-0.11	-0.31	-0.17	0.00	-0.12	0.02
X44	-0.03	0.78	-0.23	0.36	0.26	-0.35	0.04
X45	0.91	-0.21	-0.18	-0.16	0.15	-0.12	0.04
X46	0.26	0.15	0.83	0.10	-0.06	0.15	0.12
X47	-0.27	0.38	-0.24	0.51	0.52	-0.37	-0.20
X48	-0.21	-0.07	0.05	-0.27	0.28	-0.14	-0.07
X49	-0.32	0.45	0.33	0.65	0.15	0.30	0.03
X50	0.52	0.74	0.14	0.22	-0.08	0.22	0.12
X51	-0.35	0.37	-0.17	0.45	0.49	-0.29	-0.25
X52	0.31	-0.41	0.08	0.75	-0.02	-0.15	0.25
X53	0.25	0.66	0.29	0.27	-0.33	0.12	-0.23
X54	0.78	-0.52	0.08	-0.30	0.02	-0.03	-0.07
X55	-0.79	0.22	-0.28	0.06	-0.16	0.36	-0.16
X56	-0.23	-0.04	-0.23	0.55	0.03	-0.35	0.29
X57	0.82	0.45	-0.02	-0.02	0.08	-0.12	0.26
X58	0.36	0.06	0.06	-0.29	0.02	0.17	0.78
X59	-0.83	-0.04	-0.45	0.00	0.02	0.18	0.09
X60	0.80	0.46	0.16	0.22	-0.26	0.04	-0.10
X61	-0.58	0.25	0.72	-0.03	0.13	0.12	0.04
X62	0.35	0.83	-0.11	0.03	0.17	0.30	0.13
X63	0.15	0.83	-0.48	0.02	0.11	-0.08	-0.13
X64	0.57	0.68	-0.31	0.23	-0.16	0.02	-0.08
X65	0.12	-0.43	0.71	0.21	-0.14	-0.02	-0.20
X66	0.05	0.82	-0.19	0.22	-0.04	-0.38	-0.10
X67	0.53	-0.15	0.05	0.62	-0.34	0.18	0.16
X68	0.88	0.00	0.03	0.25	-0.24	0.14	0.12
X69	0.72	-0.66	0.07	-0.09	-0.02	-0.15	-0.03
X70	0.25	-0.32	-0.44	0.70	-0.14	-0.05	-0.11
X71	0.40	-0.83	-0.15	0.26	-0.04	-0.10	-0.08
X72	-0.29	0.54	-0.68	-0.13	0.14	-0.05	0.03
X73	-0.32	-0.36	-0.29	0.66	-0.11	0.11	-0.05
X74	0.57	-0.69	0.29	-0.15	-0.13	0.04	0.08
X75	0.04	0.19	-0.77	0.46	-0.08	0.32	-0.05
X76	0.77	0.47	-0.05	-0.13	0.18	-0.02	0.29
X77	-0.87	0.04	-0.33	-0.20	-0.16	0.04	0.06
X78	-0.15	-0.03	0.69	0.38	-0.06	0.23	0.32
X79	-0.71	0.40	0.34	-0.13	0.15	-0.25	-0.15
X80	0.46	-0.52	0.61	-0.06	-0.21	-0.08	0.16
X81	0.52	0.71	-0.02	0.02	-0.20	-0.28	0.16
X82	0.17	-0.02	0.08	0.62	-0.44	0.33	0.06
X83	-0.64	-0.24	-0.30	0.22	-0.07	0.46	0.17
X84	0.67	0.42	-0.39	-0.25	-0.11	0.12	0.02

X85	0.72	0.33	-0.18	0.02	-0.36	-0.30	0.32
X86	-0.11	0.02	0.66	0.18	-0.55	-0.19	-0.23
X87	0.00	0.34	0.27	0.55	-0.56	-0.22	0.13
X88	0.73	0.55	-0.09	-0.25	-0.12	0.23	-0.09
X89	0.71	-0.31	-0.18	-0.13	0.01	0.31	-0.33
X90	-0.63	0.35	-0.27	-0.18	0.25	-0.36	0.12
X91	-0.34	-0.08	-0.41	0.37	0.36	0.28	-0.03
X92	0.11	-0.72	0.14	0.08	-0.17	0.25	0.36
X93	-0.31	-0.19	-0.46	0.08	-0.28	0.12	0.53
X94	0.41	0.69	-0.05	0.26	0.32	-0.12	0.33
X95	-0.31	0.39	-0.04	0.05	0.51	-0.34	-0.02
X96	-0.43	-0.03	-0.60	0.27	-0.12	0.02	0.16
X97	0.14	0.09	0.42	0.15	-0.70	-0.33	-0.04
X98	0.06	-0.39	-0.03	0.24	0.38	-0.20	-0.34
X99	-0.35	-0.03	-0.22	0.27	0.43	0.24	-0.14
X100	-0.29	-0.09	-0.09	0.60	0.11	0.24	-0.46
X101	-0.52	-0.05	-0.30	-0.05	0.06	0.17	0.45
X102	0.39	0.56	-0.04	-0.17	-0.52	0.31	0.07
X103	-0.39	-0.30	0.11	-0.13	0.41	0.03	0.13
X104	0.86	0.35	-0.24	-0.22	0.02	0.01	0.10
X105	0.64	0.73	-0.17	0.05	0.09	0.13	0.02
X106	0.61	0.61	0.11	0.26	0.03	-0.27	-0.07
X107	0.59	0.37	0.05	0.24	-0.20	-0.41	-0.33
X108	0.74	0.58	0.12	0.12	0.04	0.12	-0.08
X109	0.66	0.25	-0.20	-0.19	-0.29	-0.21	-0.19
X110	-0.72	0.11	-0.14	-0.27	0.56	0.07	-0.02
X111	0.61	0.13	0.22	0.20	0.26	-0.44	0.41
X112	0.81	0.48	-0.05	-0.16	-0.05	-0.06	0.02
X113	0.43	0.61	0.22	0.18	0.03	-0.48	0.01
X114	0.67	0.25	-0.11	-0.21	-0.20	0.21	-0.41
X115	-0.68	0.13	-0.04	-0.33	0.51	0.07	0.13

PC, principal component. Xn is the number of volatile substances. Details are in table S1.

Supplemental Table S4 The primers used in this study

Gene name	Primers	Sequence（5'to 3'）
Actin	*Actin-F*	ttgctgaccgtatgagcaag
	Actin-R	ggacaatggatggaccagac
PSY1	*PSY1-F*	tggcccaaacgcatcatata
	PSY1-R	caccatcgagcatgtcaaatg
CRTISO	*CRTISO -F*	ttttggcggaatcaactacc
	CRTISO -R	gaaagcttcactcccacagc
CYCB	*CYCB -F*	tgttattgaggaagagaaatgtgtgat
	CYCB -R	tcccaccaatagccataacatttt
CEL2	*CEL2-F*	gcattacacgacggtgcttc
	CEL2-R	tcactccatctctcgccgta
CESA6	*CESA6-F*	cctgctgcccttaactcaga
	CESA6-R	gcctccatgcttaaccacct
PL	*PL-F*	ttggtggaagtgctgatccc
	PL-R	ttccattcactctccggtgc
EXP	*EXP-F*	gccaaatgacaatggtggct
	EXP-R	ccttgctttcggcatgggat

Actin was used as reference gene in present study.
